# Deep Brain Stimulation of the Nucleus Accumbens Core Affects Trait Impulsivity in a Baseline-Dependent Manner

**DOI:** 10.3389/fnbeh.2017.00052

**Published:** 2017-03-23

**Authors:** Maria C. Schippers, Bastiaan Bruinsma, Mathijs Gaastra, Tanja I. Mesman, Damiaan Denys, Taco J. De Vries, Tommy Pattij

**Affiliations:** ^1^Amsterdam Neuroscience, Department of Anatomy and Neurosciences, VU University Medical CenterAmsterdam, Netherlands; ^2^Amsterdam Neuroscience, Department of Psychiatry, Academic Medical Center, University of AmsterdamAmsterdam, Netherlands

**Keywords:** impulsivity, 5-choice serial reaction time task, delayed reward task, impulsive action, impulsive choice, deep brain stimulation, nucleus accumbens

## Abstract

Deep brain stimulation (DBS) of the nucleus accumbens (NA) is explored as a treatment for refractory psychiatric disorders, such as obsessive-compulsive disorder (OCD), depressive disorder (MDD), and substance use disorder (SUD). A common feature of some of these disorders is pathological impulsivity. Here, the effects of NAcore DBS on impulsive choice and impulsive action, two distinct forms of impulsive behavior, were investigated in translational animal tasks, the delayed reward task (DRT) and five-choice serial reaction time task (5-CSRTT), respectively. In both tasks, the effects of NAcore DBS were negatively correlated with baseline impulsive behavior, with more pronounced effects in the 5-CSRTT. To further examine the effects of DBS on trait impulsive action, rats were screened for high (HI) and low (LI) impulsive responding in the 5-CSRTT. NAcore DBS decreased impulsive, premature responding in HI rats under conventional conditions. However, upon challenged conditions to increase impulsive responding, NAcore DBS did not alter impulsivity. These results strongly suggest a baseline-dependent effect of DBS on impulsivity, which is in line with clinical observations.

## Introduction

Deep brain stimulation (DBS) is currently utilized as clinical intervention for obsessive-compulsive disorder (OCD) (Hamani et al., 2014; Van Westen et al., 2015), and explored for substance use disorder (SUD) (Luigjes et al., 2012; Pierce and Vassoler, 2013), major depressive disorder (Schlaepfer et al., 2014) and anorexia nervosa (Oudijn et al., 2013). DBS is a neurosurgical procedure in which implanted electrodes deliver electrical pulses to specific brain targets. Despite the clinical application of DBS, its underlying mechanisms of action are still poorly understood (Florence et al., 2016).

In SUD and OCD, the nucleus accumbens (NA) is often target region of DBS and known to play an important role in motivation and impulsive behavior (Cardinal et al., 2002; Cardinal, 2006; Meredith et al., 2008; Pattij and Vanderschuren, 2008). Moreover, whereas both SUD and OCD are characterized by compulsive behavior, impulsivity appears to be differentially effected in these disorders (Figee et al., 2016). For instance, whereas translational preclinical studies as well as clinical studies have shown beneficial effects of NA DBS on compulsivity (Van Kuyck et al., 2008; Denys et al., 2010; Figee et al., 2013; Kohl et al., 2014), the effects of DBS on impulsivity are less well documented and explored (Sesia et al., 2008, 2010).

It is widely recognized that impulsivity is a multi-faceted phenomenon (Evenden, 1999; Dalley et al., 2011). Impulsive action is described as diminished inhibitory control over inappropriate responses, whereas impulsive choice oftentimes is operationalized as the preference for small immediate reinforcement over large delayed reinforcement. These different forms of impulsivity have partly distinct underlying neural correlates, including differential NA involvement (Pattij and Vanderschuren, 2008; Basar et al., 2010; Winstanley, 2011). Moreover, it is well-established that within the NA there is functional compartmentalization (Groenewegen et al., 1999). In this respect, lesions of the NAcore region primarily affect impulsive choice (Cardinal et al., 2001; Pothuizen et al., 2005; Bezzina et al., 2007) and not impulsive action (Christakou et al., 2004; Pothuizen et al., 2005; Murphy et al., 2008), whereas pharmacological modulation of the NAcore has been reported to alter impulsive action (Pattij et al., 2007; Murphy et al., 2008; Economidou et al., 2012).

Interestingly, decrements in impulsive action by NAcore DBS and, opposingly, increments in impulsive action by NAshell DBS in a simple reaction time task have been reported recently (Sesia et al., 2008), yet later work failed to replicate this (Sesia et al., 2010). Moreover, two clinical case reports in OCD demonstrated that, in addition to ameliorating OCD symptoms, NA DBS also increased impulsivity and impulsive aggression (Malone et al., 2009; Luigjes et al., 2011). Collectively, these observations suggest direct DBS effects on impulsivity. A better understanding of the effects of DBS on different forms of impulsivity is highly relevant to improve the treatment potential of DBS and to better understand its mechanisms of action.

To address this, we studied the effects of NA DBS in two translational rat models measuring different forms of impulsivity, namely the five-choice serial reaction time task (5-CSRTT) to measure impulsive action (Robbins, 2002) and the delayed reward task (DRT) to assess impulsive choice (Cardinal, 2006). Outbred rats were used not representing a model of disorder, in order to further unravel the direct effects of NA DBS on impulsive behavior. In particular, DBS was applied to the core region of the NA since previous studies provided strong evidence for this specific subregion in impulsivity (Cardinal et al., 2001; Pothuizen et al., 2005; Basar et al., 2010; Dalley et al., 2011; Feja et al., 2014). Since we found baseline-dependent effects of NAcore DBS on impulsive action, in further experiments a large cohort of rats was trained in the 5-CSRTT to examine the effects of NAcore DBS in high and low trait impulsive individuals.

## Materials and methods

### Animals

Male Wistar rats (*N* = 96 in total) weighing approximately 300 grams at start of experiments were obtained from Harlan CPB (Horst, The Netherlands) and were housed in pairs until implantation of DBS electrodes. Animals were kept under a reversed light/dark cycle (lights on 7 p.m. until 7 a.m.) at controlled room temperature (21 ± 2°C) and relative humidity of 60 ± 15%. Experiments were conducted during the dark phase of the light–dark cycle. Animals were tested once daily from Monday to Friday. During training and testing phases, rats were food-restricted to 90% of their free-feeding bodyweight. Water was available *ad libitum* during the entire experiment. All experiments were approved by the Animal Care Committee of the VU University and VU University Medical Center, Amsterdam.

### Behavioral tasks

#### Apparatus

Both behavioral tasks were conducted in sixteen identical operant chambers (Med Associates Inc., St. Albans, USA) in sound-attenuating ventilated cubicles. One wall contained an array of 5 nose poke holes which could be illuminated and had an infrared beam for nose poke detection. On the opposite wall, a food magazine was situated, where the reward (45 mg precision pellets, BioServ, Frenchtown, USA) could be delivered. A white house light was situated on the same wall as the food magazine.

#### Delayed reward task

The DRT as employed in our laboratory has been described previously (Van Gaalen et al., 2006b). Briefly, after trial initiation through a nose poke into the central nose poke unit, the animals had free choice between responding into the left adjacent or right adjacent nose poke unit which were both illuminated. Poking into one unit resulted in the immediate delivery of a small reinforcer (1 food pellet), whereas poking into the other unit resulted in the delivery of a large delayed reinforcer (4 food pellets). Delays for the large reinforcer progressively increased within a session per block of 12 trials. The behavioral measure to assess task performance, i.e., the percentage preference for the large reinforcer as a function of delay, was calculated as the number of choices for the large reinforcer/(number of choices for large + small reinforcer)^*^100. Furthermore, hyperbolic curves for the percentage preference for the large reward were fitted on the individual data by the equation *V* = *A*/(1+ *kD*); where *V* is the preference for the large reward after a delay of *D* in seconds, *A* is the preference for the large reward at *D* = 0 s and *k* describes the steepness of the discounting curve (Mazur, 2006). Based on the estimated hyperbolic curve, the indifference point, the delay for which the rats switched their preference over to the immediate, small reward (i.e., the delay on which the preference for large reward <50%) was calculated.

#### Five-choice serial reaction time task

A description of the 5-CSRTT behavioral procedure in our laboratory has been described previously (Van Gaalen et al., 2006b; Wiskerke et al., 2011). Rats were trained to respond to a visual stimulus presented in one of the five nose poke units. Each session terminated after 100 trials or 30 min, whichever occurred first. Correct responses, during 1 s stimulus duration or a 2-s limited hold period, were rewarded with delivery of one food pellet. Two measures of inhibitory control were calculated, the number of premature responses and the total number of perseverative responses after a correct trial, a presumable measure for compulsivity. Premature responses during the 5 s intertrial interval (ITI) were punished by a 5-s time-out period, during which the house light was switched off. The ITI was fixed at 5 s during training, whereas during testing either a fixed ITI of 5 s or variable ITI of 5, 7, and 9 s was used. Perseverative responding after correct responses were recorded, but were without programmed consequences. Stable baseline performance was defined as >80% accuracy and <20% omissions.

### Surgery

Following stable baseline performance, rats were surgically equipped with DBS electrodes. For this purpose, prior to surgery, rats were subcutaneously injected with 5 mg/kg Ketofen 1% and 8.33 mg/kg Baytril 2.5%. DBS electrodes combined with a guide cannula (Plastics One, Germany) were bilaterally implanted in the NAcore region (coordinates: 2.3 mm rostral to bregma, 7.4 mm ventral to dura, 2.7 mm lateral to midline under an angle of 8° relative to the midline sagittal plane) under isoflurane inhalation anesthesia (±2%). DBS electrodes were anchored to the skull with stainless steel screws and dental acrylic cement. Experiments started following 1 week of recovery.

### Deep brain stimulation

Deep brain stimulation was performed with a digital stimulator (model DS8000, World Precision Instruments, Israel) and stimulus isolator (model DLS100) connected to a 4 channel commutator (Plastics One). During habituation and stimulation sessions, the electrode implants were attached to stimulation cables, which were connected to the commutator. Stimulation intensities varied across experiments between 35, 75, and 100 μA (130 Hz, biphasic square pulses, 60 μs pulse width, 200 μs zero time), which are estimated to primarily activate nerve fibers within 0.5 to 1 mm radius (Ranck, 1975; McIntyre and Grill, 2001). These stimulation parameters are comparable to previously reported DBS rat studies (Darbaky et al., 2003; Baunez et al., 2007; Sesia et al., 2008, 2010; Tan et al., 2010; Van Der Plasse et al., 2012). Sham stimulation was applied by attaching the rats to DBS cables without stimulation. Stimulation always started 5 min before session onset. DBS tests were conducted on Wednesdays and Fridays with baseline training sessions on other weekdays, during which rats were attached to DBS cables to maintain habituation to the procedure. Each rat was stimulated once a week with sham stimulation on the other test day and received every stimulation intensity once per test condition, to avoid potential carry-over effects of stimulation.

### Experimental design

#### Experiment 1: DRT

To examine the effects of NAcore DBS on impulsive choice, sixteen rats were bilaterally stimulated during the entire DRT test session with 0/35/75/100 μA DBS in a within-subjects Latin-square design (Table 1).

**Table 1:** **Experimental design**.

	**n**	**Task**	**DBS (μA)**	**Drug challenge**	**Test design**
Experiment 1	16	DRT	0, 35, 75, 100	–	Latin-square
Experiment 2	16	5-CSRTT, fixed ITI	0, 35, 75, 100	–	Latin-square
Experiment 3A	16 HI, 16 LI	5-CSRTT, fixed ITI	0, 75	–	Randomized within-subjects
		5-CSRTT, variable ITI	0, 35, 75, 100	–	Latin-square
Experiment 3B	16 MI	5-CSRTT, fixed ITI	0, 75	Saline/amphetamine	Latin-square

#### Experiment 2: 5-CSRTT

A separate group of 16 rats was tested during 5-CSRTT sessions with a fixed ITI of 5 s to study effects of NAcore DBS on impulsive action. Rats were bilaterally stimulated with 0/35/75/100 μA DBS during the entire session in a within-subjects Latin-square design (Table 1).

#### Experiment 3: trait high and low impulsive rats in the 5-CSRTT

**Figure 1:**
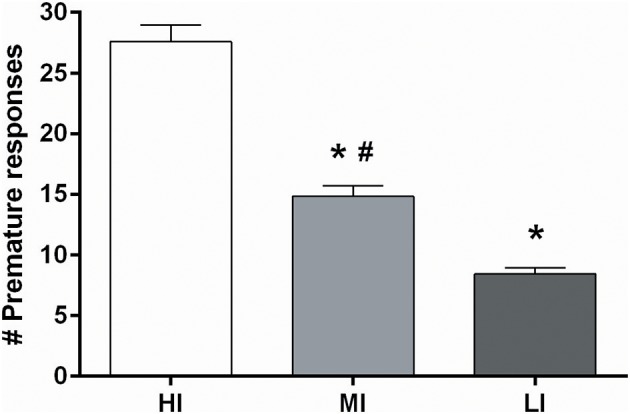
**Average of the number of premature responses on three baseline days before DBS electrodes implantation under fixed ITI conditions for all four impulsivity groups**. HI, high impulsive (*n* = 16); LI, low impulsive group (*n* = 16) (Experiment 3A), (*n* = 16); MI, moderate impulsive group (Experiment 3B) (*n* = 16). ^*^p < 0.001 compared to HI, ^#^*p* ≤ 0.001 compared to LI.

Based on the more profound baseline-dependent effect in the 5-CSRTT compared to the DRT, effects of NAcore DBS on high and low impulsive action were examined in a large cohort of 64 rats. Upon stable baseline task performance rats were divided over four quartiles based on the number of premature responses. The upper and lower quartiles were assigned as high impulsive (HI) and low impulsive (LI) rats, respectively. Rats belonging to the middle quartiles were assigned to two moderate impulsive (MI) groups, matched for their behavioral performance (Figure 1).

#### Experiment 3A: trait high and low impulsive rats in the 5-CSRTT with fixed ITI and variable ITI

First, to examine the effects of NAcore DBS on impulsive action in HI and LI rats, subjects were tested in the 5-CSRTT under a standard fixed 5 s ITI. Based on the significant baseline-dependent effects on premature responses with 75 μA in experiment 2, rats were bilaterally stimulated with 0 or 75 μA using a randomized within-subjects design. Subsequently, to increase stimulus unpredictability, HI and LI rats were also tested under variable ITI conditions. As such, ITI duration (5, 7, or 9 s) was pseudorandomly selected and all durations were equally presented during a session. Rats were bilaterally stimulated with 0/35/75/100 μA using a Latin-square within-subjects design (Table 1).

#### Experiment 3B: acute amphetamine challenges in moderate impulsive rats

To examine whether effects of NAcore DBS on impulsive action are dependent on state impulsivity, one group of MI rats was tested in the 5-CSRTT following an amphetamine dose known to robustly increase impulsive action (Van Gaalen et al., 2006a; Pattij et al., 2007; Wiskerke et al., 2011). For this, rats were tested under standard baseline conditions in the 5-CSRTT 1-s. They were injected with saline or amphetamine [(+)-Amphetamine sulfate (O.P.G. Utrecht, The Netherlands) dissolved in sterile saline, 0.5 mg/kg, i.p.] 20 min prior to testing and bilaterally stimulated with 0 and 75 μA in a Latin-square within-subjects design (Table 1).

### Electrode placement verification

After the last test day, rats were deeply anesthetized with Euthasol (AST Farma, The Netherlands; i.p.) and perfused transcardially with 100 ml 0.9% NaCl, followed by 500 ml 4% paraformaldehyde in 0.1 M PBS. Brains were removed, post-fixed in the same fixative for 24 h and cryoprotected in 30% sucrose. Coronal 40 μm sections were cut on a sliding microtome and stained with cresyl violet for electrode placement verification.

### Statistical analyses

Data are presented as means ± s.e.m. and were analyzed using IBM SPSS Statistics 20.0 (IBM, New York, USA). Dependent variables in the DRT (experiment 1) were percentage choice for the large reward, omitted trial starts, omitted choice trials, omitted forced trials, ITI responses, latencies and indifference point. In the 5-CSRTT (experiments 2 and 3), dependent variables were premature responses, perseverative responses after correct choice, accurate choice, omissions, correct response latency and feeder latency. In both experiments, repeated measures ANOVAs were performed with DBS intensity as within-subjects factor. Moreover, in experiment 3A, repeated measures ANOVA with impulsivity group as between-subjects variable and DBS intensity and ITI duration for variable ITI experiments as within-subjects variables. In experiment 3B, data were analyzed using repeated measures ANOVA with DBS intensity and treatment (saline vs. amphetamine), or ITI duration (5, 7, and 9 s) as within-subjects variables.

Normal distribution of data was tested with the Shapiro-Wilk test. In case variables were not normally distributed, data were transformed using a Log10 transformation. In all repeated measures ANOVAs, degrees of freedom were corrected with Huyn-Feldt corrections in case Mauchly's test was significant and sphericity assumptions were violated. In case of statistical significant main effects, further *post-hoc* tests were performed with Bonferonni corrections for multiple comparisons. Correlations were performed using a two-tailed Pearson's correlation. Statistical significance was set at *p* < 0.05 for all analyses.

## Results

### Histology and exclusion of rats

**Figure 2:**
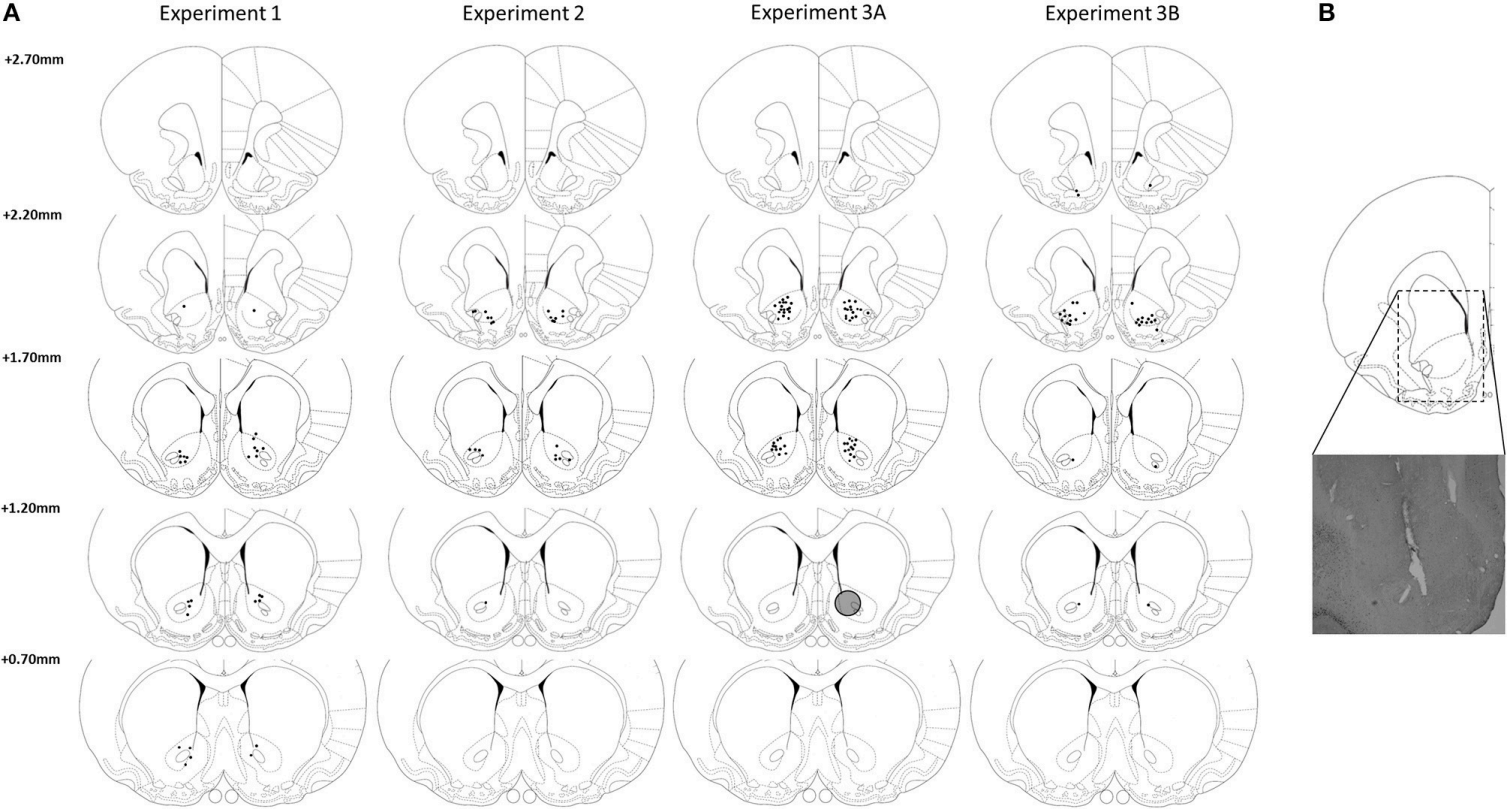
**(A)** Verification of DBS electrodes placement in the NAcore at the level of 2.70, 2.20, 1.70, and 0.70 mm rostral to bregma. Rats with placements of DBS electrodes at 2.70 mm rostral to bregma were excluded from analysis. Gray circle depicts estimated maximal stimulation area around an electrode tip (Ranck, 1975; McIntyre and Grill, 2001). **(B)** Representative electrode placement in the NAcore. Drawings are adapted from Paxinos and Watson (1998).

As depicted in Figure 2, most DBS electrodes were positioned in the NAcore at the level of 2.20 and 1.70 mm rostral to bregma. In experiment 1, one rat was excluded from analyses, because histological inspection revealed a large infection around the electrode tip, resulting in *n* = 15 subjects. In experiment 2, one rat was excluded from analyses due to technical problems during test days and three rats died unexpectedly during the experimental phase, resulting in *n* = 12 subjects for this experiment. In experiment 3A, two rats (both from LI group) were excluded due to high numbers of omissions during training days (on average 53.9 ± 1.6 omissions per day). Five rats (three HI and two LI) were excluded, because their post-surgery level of premature responses had changed compared to pre-surgery levels to such extend that they did not meet the requirements for trait impulsivity anymore. Three HI rats were excluded due to early electrode loss. Therefore, in total, 12 LI rats and 11 HI rats were included in the analyses of the fixed ITI experiments and 12 LI rats and 10 HI rats were included in the variable ITI experiments. In experiment 3B, three rats had misplaced DBS electrodes and were therefore excluded from all analyses, resulting in *n* = 13 subjects.

### Experiment 1: DRT

**Figure 3:**
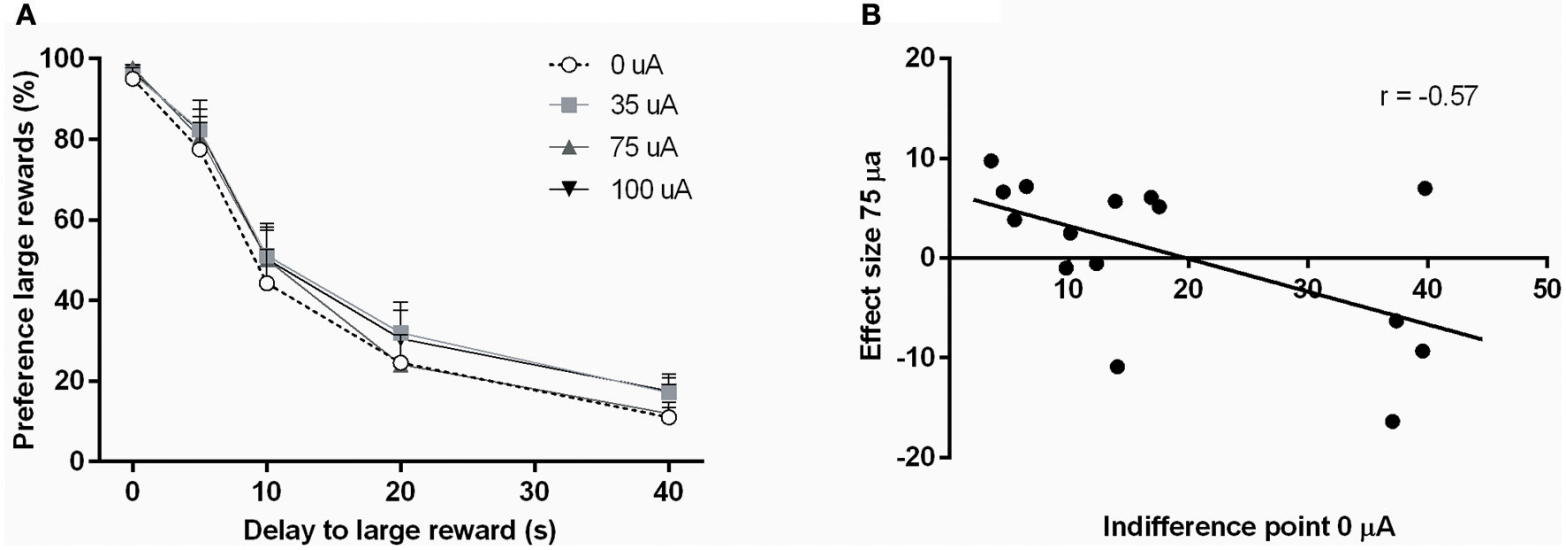
**(A)** Effects of different DBS stimulation intensities (0, 35, 75, and 100 μA) on the percentage preference for the large reward in the DRT. **(B)** Correlation between the baseline indifference point under baseline condition (0 μA) and the effect of DBS at 75 μA.

In the DRT, the preference for the large reward decreased significantly with increasing delays [Delay: *F*_(4, 56)_ = 109.33, ε = 0.47, *p* < 0.001]. This was not altered by NAcore DBS [DBS: *F*_(3, 42)_ = 1.39, N.S.; DBS^*^Delay: *F*_(12, 168)_ = 0.31, ε = 0.44, N.S.; Figure 3A]. In addition, the indifference point was not altered by NAcore DBS [*F*_(3, 42)_ = 1.95, N.S.]. Nonetheless, further in-depth analyses revealed a significant negative correlation between indifference point and the magnitude of effect of 75 μA NAcore DBS (*r* = −0.57, *p* = 0.028) (Figure 3B), indicating baseline-dependent effects of DBS on impulsive choice. The other stimulation intensities did not show significant correlations between indifference points and effect size of DBS (35 μA vs. 0 μA *r* = −0.09, N.S.; 100 μA vs. 0 μA *r* = 0.02, N.S.). Other task parameters, such as omitted starts of a trial [*F*_(3, 42)_ = 0.01, N.S.], omitted choice trials [*F*_(3, 42)_ = 0.58, N.S.], omitted forced trials [*F*_(3, 42)_ = 1.18, ε = 0.63, N.S.], ITI responses [*F*_(3, 42)_ = 0.70, ε = 0.58, N.S.], latency to start a trial [*F*_(3, 42)_ = 0.97, N.S.], latency to collect a small reward [*F*_(3, 42)_ = 1.30, N.S.] and latency to collect a large reward [*F*_(3, 42)_ = 1.28, N.S.] were not affected by NAcore DBS (Table 2).

**Table 2:** **Effects of DBS on auxiliary measures in the DRT (Experiment 1)**.

	**DBS**			
	**0 μA**	**35 μA**	**75 μA**	**100 μA**
ITI pokes (#)	227.0 ± 66.9	243.7 ± 72.7	189.9 ± 43.5	215.7 ± 46.2
Omission start (#)	5.3 ± 1.0	5.2 ± 1.0	5.2 ± 1.1	5.3 ± 1.1
Omission choice (#)	4.5 ± 0.9	4.5 ± 0.8	5.5 ± 1.1	4.5 ± 0.9
Omission forced trials (#)	3.3 ± 0.3	2.4 ± 0.4	3.9 ± 0.7	3.7 ± 0.8
latency small reward (s)	0.83 ± 0.05	0.86 ± 0.04	0.92 ± 0.07	0.90 ± 0.05
latency large reward (s)	0.91 ± 0.06	0.94 ± 0.08	1.06 ± 0.09	0.92 ± 0.08
latency start trial (s)	2.32 ± 0.15	2.13 ± 0.12	2.37 ± 0.18	2.23 ± 0.15

### Experiment 2: 5-CSRTT

**Figure 4:**
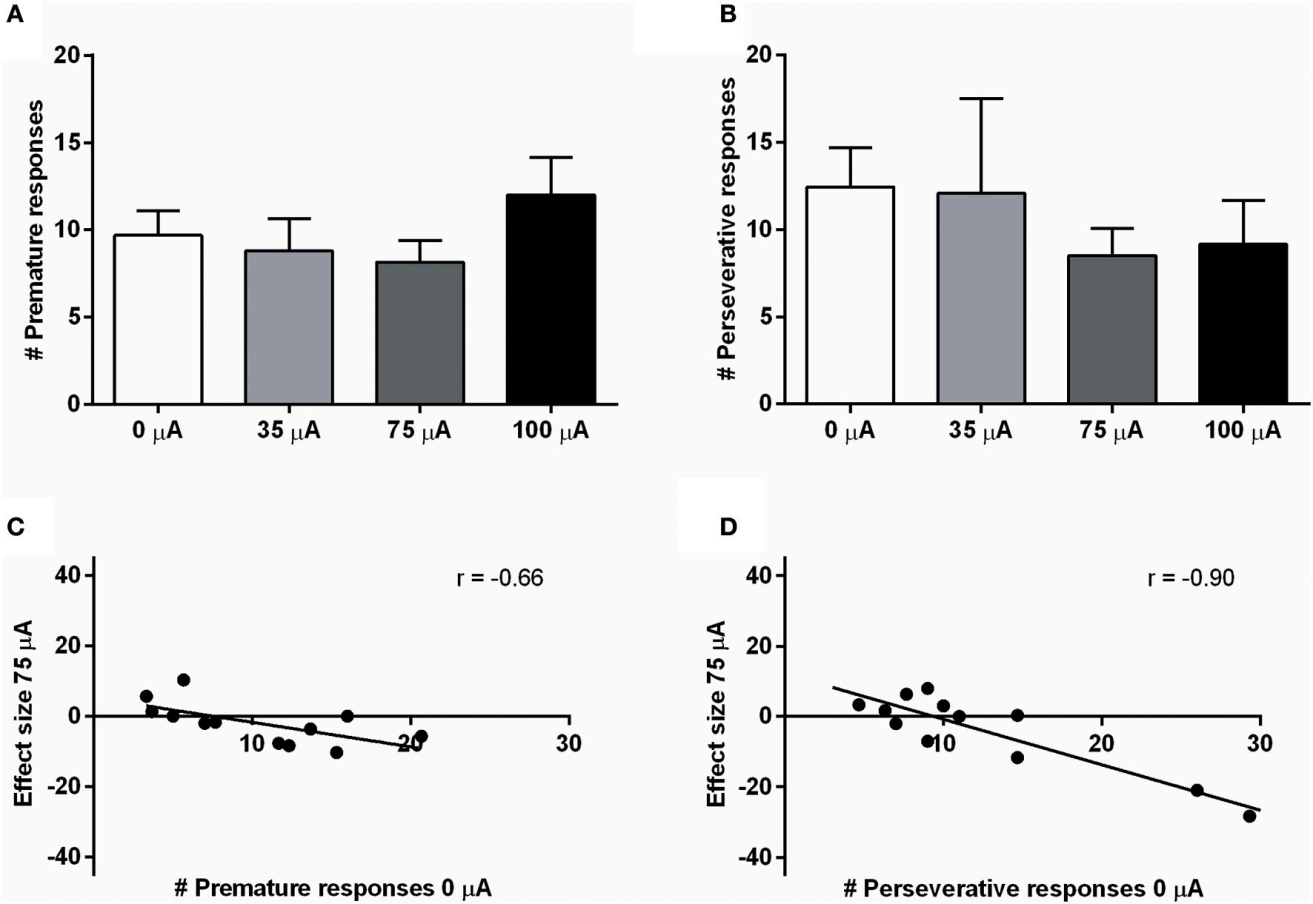
**(A)** Effects of different DBS stimulation intensities (0, 35, 75, and 100 μA) on the number of premature responses in the 5-CSRTT. (**B**) Effects of different DBS stimulation intensities (0, 35, 75, and 100 μA) on the number of perseverative responses after a correct choice. (**C**) Negative correlation between baseline premature responding (0 μA) and the effect of 75 μA DBS. (**D**) Negative correlation between baseline perseverative responding after a correct choice (0 μA) and the effect of 75 μA DBS.

NAcore DBS had no significant effects on overall performance in the 5-CSRTT. The main measure of impulsive action, premature responses, was unaffected by the different DBS intensities [*F*_(3, 33)_ = 0.99, N.S.; Figure 4A]. To explore whether the effects of DBS were baseline-dependent, correlation analyses on baseline premature responses and DBS effect size were performed. These analyses revealed a significant negative correlation for 35 μA (*r* = −0.67, *p* = 0.017), 75 μA (*r* = −0.66, *p* = 0.021; Figure 4C), but not for 100 μA (100 μA *r* = −0.28, N.S.). Similar to premature responding, overall perseverative responding after correct choice was not altered by NAcore DBS [*F*_(3, 33)_ = 1.01, N.S.; Figure 4B], yet correlation analyses revealed a significant negative correlation between baseline perseverative responses and the effect size of 75 μA (*r* = −0.90, *p* < 0.001; Figure 4D) and 100 μA (*r* = −0.77, *p* = 0.004), but not 35 μA (*r* = 0.16, N.S.). Further correlational analyses between baseline premature responding and baseline perseverative responding after correct choice revealed no significant relation between these parameters (*r* = 0.036, N.S.), suggesting that the baseline-dependent effect of NAcore DBS on perseverative responding is independent of baseline impulsivity. Other task parameters in the 5-CSRTT, such as accurate choice [*F*_(3, 33)_ = 1.27, N.S.], number of omitted trials [*F*_(3, 33)_ = 0.991, ε = 0.46, N.S.], latency to respond correctly [*F*_(3, 33)_ = 1.01, ε = 0.38, N.S.] and latency to collect a reward [*F*_(3, 33)_ = 1.09, ε = 0.56, N.S.] were not changed by NAcore DBS (Table 3).

**Table 3:** **Effects of DBS on measures of attention and motivation in the 5-CSRTT under fixed ITI conditions (Experiment 2)**.

	**DBS**			
	**0 μA**	**35 μA**	**75 μA**	**100 μA**
Accuracy (%)	89.3 ± 1.5	90.2 ± 2.3	90.7 ± 1.4	87.9 ± 2.0
Omissions (#)	11.3 ± 1.4	17.7 ± 5.2	13.1 ± 2.2	15.4 ± 2.1
Correct response latency (s)	0.62 ± 0.02	0.72 ± 0.09	0.63 ± 0.02	0.65 ± 0.02
Feeder latency (s)	3.62 ± 0.54	2.55 ± 0.27	2.80 ± 0.21	3.19 ± 0.62

### Experiment 3A: trait high and low impulsive rats in the 5-CSRTT with fixed ITI and variable ITI

#### Fixed ITI

**Figure 5:**
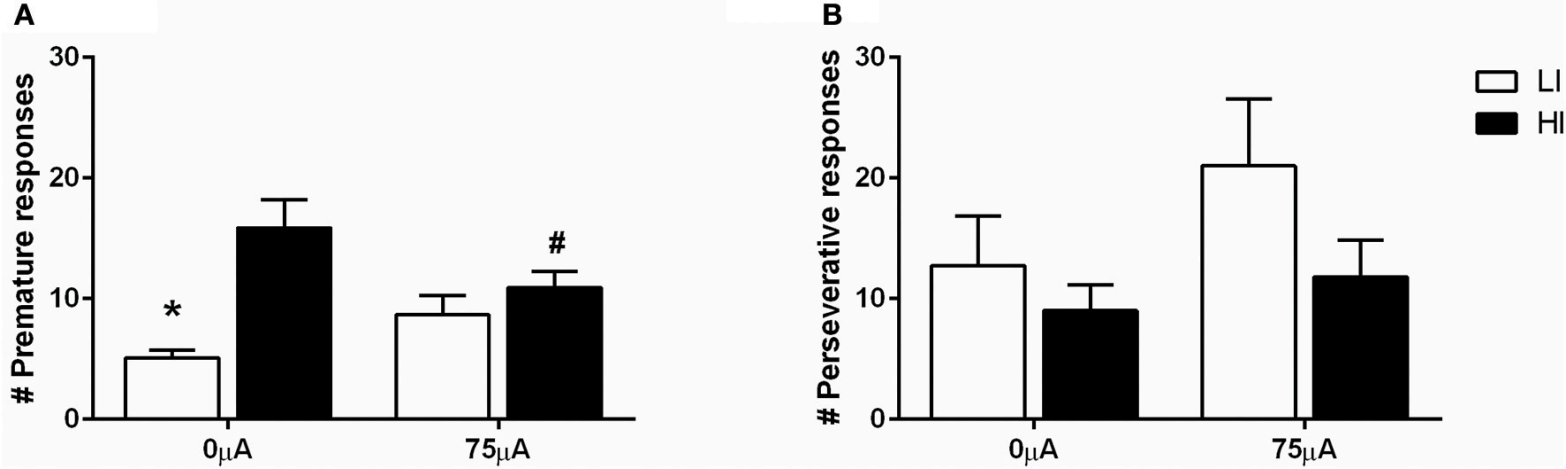
**Effects of DBS in LI (*n* = 12) and HI (*n* = 11) rats in the 5-CSRTT under fixed ITI condition on (A)** the number of premature responses and **(B)** the number of perseverative responses after a correct choice. ^*^p = 0.001 and ^#^*p* = 0.017 compared to HI under baseline conditions (0 μA).

DBS in the 5-CSRTT under fixed ITI conditions decreased premature responding in HI rats (Figure 5A), as revealed by repeated measures ANOVA [DBS: *F*_(1, 21)_ = 0.330, N.S.; impulsivity: *F*_(1, 21)_ = 12.359, *p* = 0.002; DBS^*^impulsivity: *F*_(1, 21)_ = 11.790, *p* = 0.002]. *Post-hoc* testing showed a significant difference between HI and LI rats under baseline conditions [0 μA: HI vs. LI *t* = 4.469 (11.591), *p* = 0.001], which was diminished by DBS [75 μA: HI vs. LI, *t* = 1.044 (21), N.S.]. This was caused by a significant decrease in premature responses in the HI group only, and failed to reach significance in the LI group [HI: 0 vs. 75 μA, *t* = 2.854 (10), *p* = 0.017; LI: 0 vs. 75 μA, *t* = −2.017 (11), *p* = 0.069].

Perseverative responding after correct choice was affected by DBS in both HI and LI rats [*F*_(1, 21)_ = 5.220, *p* = 0.033], independent of impulsivity levels [impulsivity: *F*_(1, 21)_ = 0.928, N.S.; DBS^*^impulsivity: *F*_(1, 21)_ = 0.905, N.S.; Figure 5B]. Accurate choice, a measure of visuospatial attention, was significantly higher in LI rats [impulsivity: *F*_(1, 21)_ = 8.734, *p* = 0.008], but this parameter was not affected by DBS [DBS: *F*_(1, 21)_ = 2.145, NS; DBS^*^impulsivity: *F*_(1, 21)_ = 2.511, N.S.; Table 4]. DBS differentially affected correct response latencies in HI and LI rats [DBS: *F*_(1, 21)_ = 0.017, NS; impulsivity: *F*_(1, 21)_ = 1.885, N.S.; DBS^*^impulsivity: *F*_(1, 21)_ = 4.760, *p* = 0.041]. Under baseline conditions, response latencies in LI rats were slower compared to HI rats [0 μA: HI vs. LI, *t* = −2.227 (15.85), *p* = 0.041]. DBS increased latencies in HI rats only [HI: 0 vs. 75 μA, *t* = −2.390 (10), *p* = 0.038; LI: 0 vs. 75 μA, *t* = 1.211 (11), N.S.], resulting in comparable response latencies between HI and LI rats [75 μA: HI vs. LI *t* = −0.378 (21), N.S.; Table 4]. There were no significant group differences or DBS effects on the number of omissions [DBS: *F*_(1, 21)_ = 1.903, NS; impulsivity: F_(1, 21)_ = 2.820, N.S.; DBS^*^impulsivity: *F*_(1, 21)_ = 1.199, N.S.] and feeder latencies [DBS *F*_(1, 21)_ = 0.136, NS; impulsivity: *F*_(1, 21)_ = 2.757, N.S.; DBS^*^impulsivity: *F*_(1, 21)_ = 0.917, N.S.; Table 4].

**Table 4:** **Effects of DBS on HI and LI rats on measurements of attention and motivation in the 5-CSRTT with fixed ITI duration (Experiment 3A)**.

	**DBS**	**Accuracy (%)**	**Omissions**	**Correct response latency (s)**	**Feeder latency (s)**
HI	0 μA	81.5 ± 2.6	8.9 ± 1.3	0.6 ± 0.01	2.3 ± 0.1
	75 μA	85.7 ± 2.1	7.7 ± 1.5	0.6 ± 0.02^*^	2.2 ± 0.1
LI	0 μA	90.0 ± 1.2	10.4 ± 1.0	0.7 ± 0.03^*^	2.5 ± 0.3
	75 μA	89.8 ± 1.1	10.2 ± 1.0	0.6 ± 0.02	2.8 ± 0.3

* *p < 0.05 compared to HI 0 μA*.

#### Variable ITI

**Figure 6:**
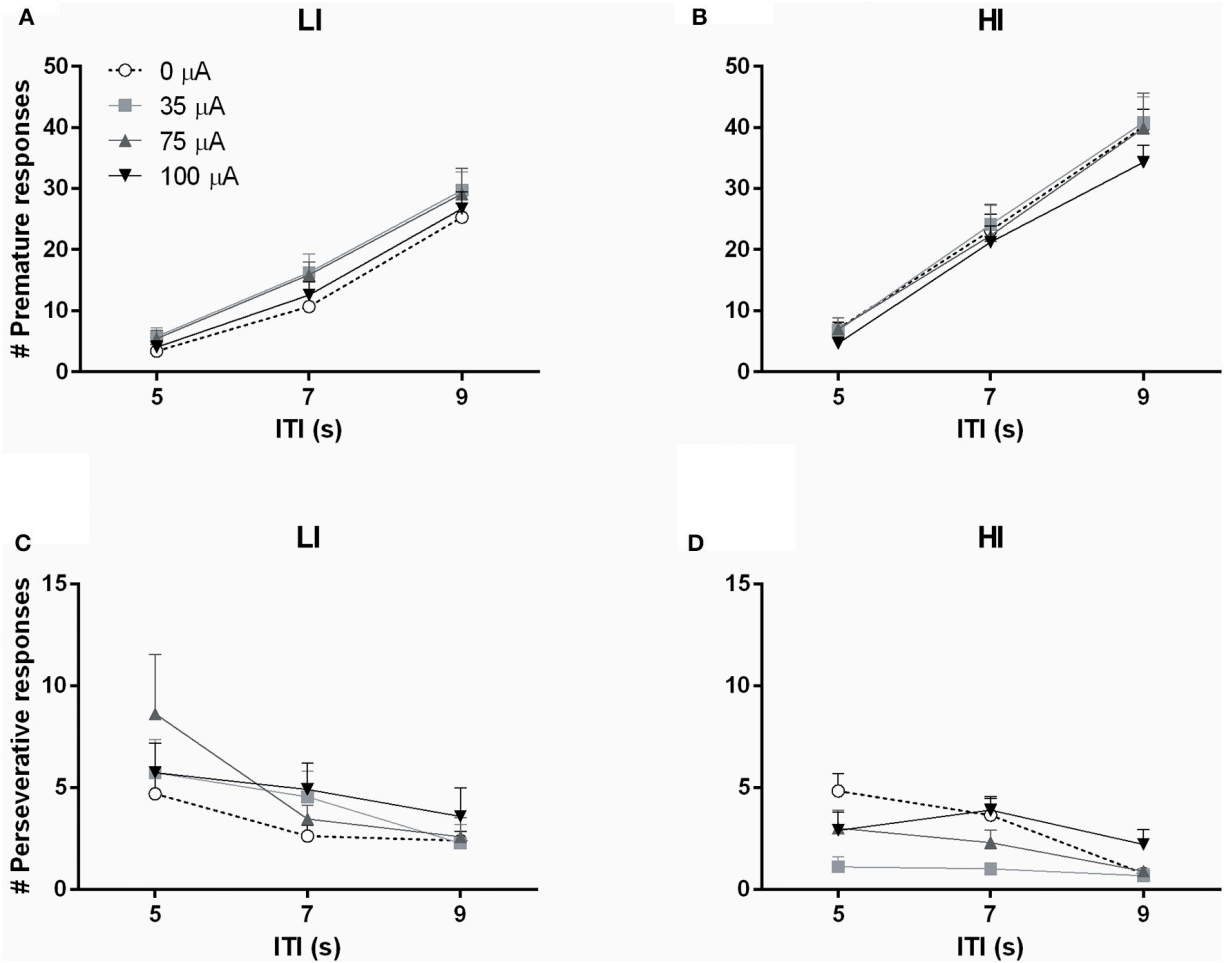
**Effects of DBS on high impulsive (HI, *n* = 10) and low impulsive (LI, *n* = 12) rats in the 5-CSRTT with variable ITI conditions expressed as (A,B)** the number of premature responses and **(C,D)** the number of perseverative responses, per ITI duration.

Subjecting rats to variable ITI conditions on test days revealed a significant difference between HI and LI rats on total number of premature responses across all ITI durations [impulsivity: *F*_(1, 20)_ = 8.933, *p* = 0.007]. This measure was not altered by DBS [DBS: *F*_(3, 60)_ = 1.367, N.S.; DBS^*^impulsivity: *F*_(3, 60)_ = 0.662, N.S.]. Analyses per ITI duration revealed significantly increased premature responding with increased ITI length, independent of baseline impulsivity [ITI: *F*_(2, 40)_ = 493.132, ε = 0.77, *p* < 0.001; ITI^*^impulsivity: *F*_(2, 40)_ = 0.789, N.S.], but these analyses revealed no effect of NAcore DBS too [DBS: *F*_(3, 60)_ = 1.164, N.S.; DBS^*^impulsivity: *F*_(3, 60)_ = 0.916, N.S.; DBS^*^impulsivity^*^ITI: *F*_(6, 120)_ = 0.761, N.S.; Figures 6A,B].

The total number of perseverative responses after correct choice, summed over all ITI durations, did not differ between HI and LI rats [*F*_(1, 20)_ = 1.472, N.S.] and was not affected by DBS [DBS: *F*_(3, 60)_ = 2.311, *p* = 0.085; DBS^*^impulsivity: *F*_(3, 60)_ = 0.272, N.S.]. However, analyses per ITI duration showed that perseverative responses were attenuated by increasing ITI length [ITI *F*_(2, 40)_ = 15.676, *p* < 0.001]. In general, LI showed a higher number of perseverative responses [group *F*_(1, 20)_ = 4.769, *p* = 0.041]. In contrast to fixed ITI conditions, analyses per ITI duration revealed a significant effect of DBS treatment on perseverative responding dependent on impulsivity group [DBS *F*_(3, 60)_ = 4.586, *p* = 0.006; DBS^*^impulsivity *F*_(3, 60)_ = 3.536, *p* = 0.020]. *Post-hoc* analyses revealed a significant decrease in HI rats stimulated with 35 μA compared to 0 μA (*p* = 0.001) and a borderline significant decrease at 75 μA (*p* = 0.059). DBS did not significantly change perseverative responding in LI rats (Figures 6C,D).

Accurate choice was significantly reduced with increasing ITI length [ITI: *F*_(2, 40)_ = 48.967, *p* < 0.001], to the same extent in HI and LI rats [impulsivity: *F*_(1, 20)_ = 3.083, N.S.; ITI^*^impulsivity: *F*_(2, 40)_ = 1.945, N.S.]. Additionally, this measure was not affected by DBS [DBS: *F*_(3, 60)_ = 2.188, N.S.; DBS^*^impulsivity: *F*_(3, 60)_ = 0.895, N.S; DBS^*^ITI^*^impulsivity: *F*_(6, 120)_ = 1.425, N.S.; Table 4]. Also the number of omissions was decreased by increasing ITI durations [ITI: *F*_(2, 40)_ = 56.523, ε = 0.740, *p* < 0.001]. There were no differences between HI and LI rats [impulsivity *F*_(1, 20)_ = 2.379, N.S.; ITI^*^impulsivity *F*_(2, 40)_ = 0.478, ε = 0.740, N.S.] and DBS increased the number of omissions to the same extent in HI and LI rats [DBS: *F*_(3, 60)_ = 5.843, ε = 0.720, *p* = 0.005; DBS^*^impulsivity: *F*_(3, 60)_ = 0.216, ε = 0.720, N.S.; DBS^*^ITI^*^impulsivity: *F*_(6, 120)_ = 0.739, ε = 0.995, N.S.]. *Post-hoc* analyses revealed a significant increase in omission errors induced by 75 μA DBS compared to 0 μA (*p* = 0.022) (Table 5). Correct response latencies were significantly decreased with increasing ITI length [ITI: *F*_(2, 40)_ = 4.670, *p* = 0.015]. There was no significant difference between LI and HI rats [impulsivity: *F*_(1, 20)_ = 3.940, N.S.], yet latencies were differentially affected in HI and LI rats depending on ITI length [ITI^*^impulsivity group: *F*_(2, 40)_ = 5.833, *p* = 0.006]. In addition, DBS treatment significantly altered correct response latencies differentially in LI and HI rats [DBS: *F*_(3, 60)_ = 3.126, *p* = 0.032; DBS^*^impulsivity: *F*_(3, 60)_ = 2.248, N.S.; DBS^*^ITI: *F*_(6, 120)_ = 6.529, *p* < 0.001; DBS^*^ITI^*^impulsivity: *F*_(6, 120)_ = 2.482, *p* = 0.027; Table 4]. Further analyses revealed that 35 μA increased correct response latencies at ITI 9s compared to ITI 5 s in the HI rats, whereas in LI rats at these conditions correct response latencies were reduced (*p* < 0.001). Likewise, in HI rats 75 μA increased correct response latencies at ITI 9s compared to ITI 5 s and not in LI rats (*p* = 0.053). Feeder latencies were higher in LI rats compared to HI rats [impulsivity: *F*_(1, 20)_ = 17.845, *p* < 0.001], but variable ITI duration or DBS treatment did not alter this parameter [ITI: *F*(2, 40) = 2.920, ε = 0.881, N.S.; DBS: *F*_(3, 60)_ = 1.711, ε = 0.835, N.S.; DBS^*^group: *F*_(3, 60)_ = 0.463, ε = 0.813, N.S.; Table 5].

**Table 5:** **Effects of DBS in HI and LI rats on behavioral performance in the 5-CSRTT under variable ITI conditions (Experiment 3A)**.

			**ITI 5**	**ITI 7**	**ITI 9**
Accurate choice (%)	HI	0 μA	86.3 ± 1.9	82.3 ± 2.2	77.8 ± 1.2
		35 μA	86.4 ± 2.0	83.0 ± 1.8	71.3 ± 3.6
		75 μA	92.9 ± 1.9	82.9 ± 2.4	81.3 ± 3.3
		100 μA	88.6 ± 2.6	80.4 ± 1.2	80.9 ± 4.3
	LI	0 μA	92.3 ± 1.1	84.8 ± 2.1	81.0 ± 2.2
		35 μA	88.6 ± 1.9	84.5 ± 1.7	80.9 ± 3.8
		75 μA	88.2 ± 1.4	86.8 ± 2.0	83.2 ± 2.3
		100 μA	88.9 ± 1.8	84.5 ± 1.8	84.4 ± 1.8
Omissions (no. per session)	HI	0 μA	5.1 ± 0.7	2.9 ± 0.7	1.5 ± 0.2
		35 μA	5.8 ± 0.8	2.9 ± 0.9	1.3 ± 0.4
		75 μA	8.1 ± 1.2	4.5 ± 1.1	3.6 ± 1.1
		100 μA	6.9 ± 1.6	1.6 ± 0.5	1.3 ± 0.3
	LI	0 μA	5.4 ± 0.6	3.5 ± 0.3	2.6 ± 0.3
		35 μA	6.4 ± 1.1	3.3 ± 0.7	2.4 ± 0.5
		75 μA	8.6 ± 0.9	4.8 ± 0.9	4.3 ± 0.6
		100 μA	6.9 ± 1.0	4.0 ± 0.7	2.9 ± 0.6
Correct response latency (s)	HI	0 μA	0.6 ± 0.02	0.7 ± 0.01	0.7 ± 0.03
		35 μA	0.6 ± 0.03	0.6 ± 0.03	0.7 ± 0.02
		75 μA	0.6 ± 0.02	0.6 ± 0.02	0.7 ± 0.05
		100 μA	0.6 ± 0.03	0.6 ± 0.02	0.5 ± 0.02
	LI	0 μA	0.7 ± 0.02	0.6 ± 0.02	0.7 ± 0.02
		35 μA	0.7 ± 0.02	0.6 ± 0.03	0.6 ± 0.03
		75 μA	0.7 ± 0.02	0.6 ± 0.02	0.7 ± 0.02
		100 μA	0.7 ± 0.02	0.7 ± 0.02	0.6 ± 0.02
Feeder latency (s)	HI	0 μA	1.9 ± 0.1	2.2 ± 0.2	1.7 ± 0.04
		35 μA	1.6 ± 0.5	1.7 ± 0.6	2.1 ± 0.3
		75 μA	1.9 ± 0.1	1.8 ± 0.1	2.2 ± 0.2
		100 μA	1.8 ± 0.1	2.1 ± 0.3	2.3 ± 0.3
	LI	0 μA	2.2 ± 0.2	2.5 ± 0.3	2.7 ± 0.3
		35 μA	2.3 ± 0.2	2.0 ± 0.1	3.0 ± 0.6
		75 μA	3.2 ± 0.4	3.0 ± 0.7	2.4 ± 0.3
		100 μA	2.8 ± 0.5	2.4 ± 0.3	4.2 ± 0.9

### Experiment 3B: acute amphetamine challenges in moderate impulsive rats

**Figure 7:**
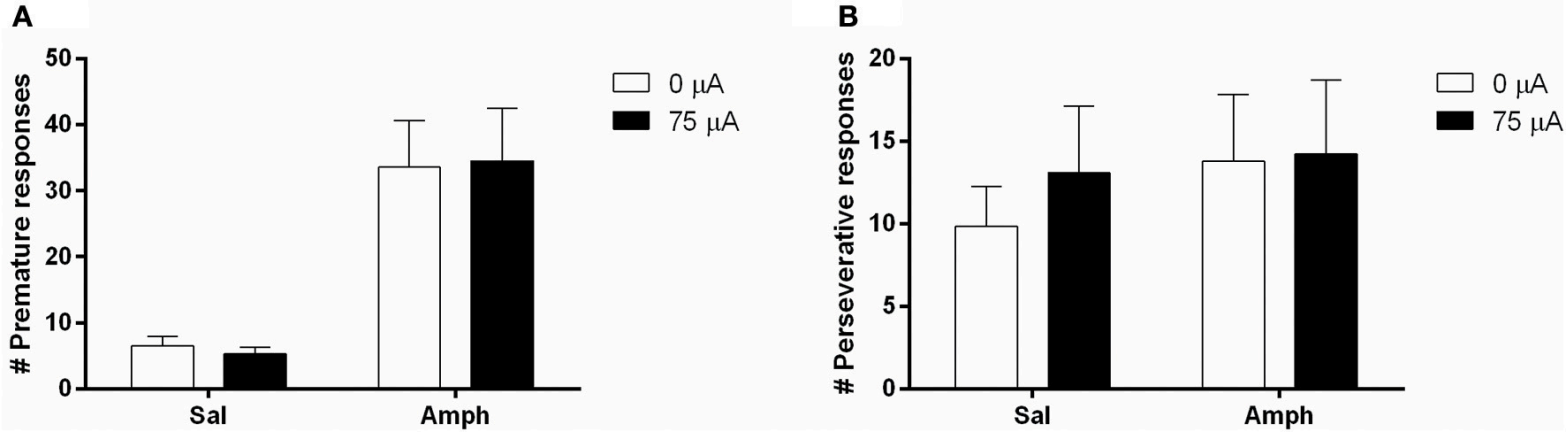
**Effects of DBS in MI rats in the 5-CSRTT after vehicle (Sal) or an acute challenge with 0.5 mg/kg amphetamine (Amph) on (A)** the number of premature responses and **(B)** the number of perseverative responses after correct choice.

Systemic 0.5 mg/kg amphetamine challenges significantly increased premature responding in MI rats [*F*_(1, 12)_ = 36.955, *p* < 0.001; Figure 7A]. The number of perseverative responses after correct choice was not altered by amphetamine [*F*_(1, 12)_ = 0.279, N.S.; Figure 7B]. DBS did not significantly change these parameters [Premature responses: DBS: *F*_(1, 12)_ = 0.008, N.S., Amph^*^DBS: *F*_(1, 12)_ = 0.108, N.S.; Perseverative responses: DBS: *F*_(1, 12)_ = 0.008, N.S., Amph^*^DBS: *F*_(1, 12)_ = 0.293, N.S.]. Amphetamine administration significantly decreased accuracy [*F*_(1, 12)_ = 25.159, *p* < 0.001] and the latency for a correct response [*F*_(1, 12)_ = 5.091, *p* = 0.043]. Other behavioral measures in the task were neither altered by the amphetamine challenge [Omissions: *F*_(1, 12)_ = 0.178, N.S.; Feeder latency: *F*_(1, 12)_ = 2.836, N.S.]. None of the auxiliary parameters were affected by DBS [DBS: *F*_(1, 12)_ < 2.339, N.S.; Amph^*^DBS: *F*_(1, 12)_ < 3.162, N.S.; Table 6].

**Table 6:** **Effects of DBS, amphetamine and their combination on measurements of attention and motivation in the 5-CSRTT in MI rats (Experiment 3B)**.

		**Saline**	**Amphetamine (0.5mg/kg)**
Accurate choice (%)	0 μA	91.1 ± 1.5	84.6 ± 2.0
	75 μA	92.4 ± 0.8	83.0 ± 1.9
Omissions (#)	0 μA	16.8 ± 3.3	14.4 ± 3.2
	75 μA	16.3 ± 3.2	17.3 ± 3.5
Correct response latency (s)	0 μA	0.6 ± 0.01	0.6 ± 0.01
	75 μA	0.7 ± 0.02	0.6 ± 0.02
Feeder latency (s)	0 μA	1.7 ± 0.2	1.9 ± 0.3
	75 μA	1.8 ± 0.2	2.9 ± 0.7

## Discussion

In the present study, we found no effect of NAcore DBS on measures of impulsive choice when analyzed on a group level. However, correlational analyses revealed a negative, baseline-dependent DBS effect, suggesting that NAcore DBS increases impulsive choice in low impulsive rats, whereas DBS attenuates impulsive choice in high impulsive rats. Similarly, NAcore DBS also exerted baseline-dependent effects on impulsive action in the 5-CSRTT. These findings fit with clinical observations in OCD and SUD patients in which effects of DBS strongly depend on baseline behavior (Heldmann et al., 2012; Figee et al., 2013), and the current data suggest appropriate face validity and clinical relevance.

Based on the profound baseline-dependent effects found in the 5-CSRTT compared to the DRT, we selected HI and LI rats from a cohort of 64 rats to further explore the effects of NAcore DBS on impulsive action. When HI and LI rats were tested under fixed ITI 5s conditions, NAcore DBS decreased premature responding specifically in HI rats, corroborating the effects found in experiment 2. However, perseverative responses after correct choice were increased by NAcore DBS in both HI and LI rats, contrasting the baseline-dependent effects on this parameter.

To date, DBS on impulsive action in rodents has only been tested in a simple reaction time paradigm, which involved pushing a magazine panel until a tone was presented at variable intervals (Sesia et al., 2008, 2010). Initially, NAcore DBS was found to decrease impulsive action (Sesia et al., 2008), however it was reported later that NAcore DBS had no effect on impulsive action, yet decreased perseverative responding. The contrasting results were presumably related to electrode position, since electrodes were located more ventrally in the latter study (Sesia et al., 2010). In our study, the ventral position of the electrodes was most comparable to the study in which they reported null effects on premature responding, yet decreased perseverative responding, similar to our results under variable ITI conditions (Sesia et al., 2010). This observation fits with recent clinical DBS observations that subtle changes in electrode position might influence different pathways and as such therapeutic outcomes, suggesting that electrode placement is very critical in reaching optimal treatment effects with DBS (Lujan et al., 2012).

Previous lesion studies have clearly highlighted the importance of the NAcore in measures of impulsive behavior in the DRT (Cardinal et al., 2001; Pothuizen et al., 2005) and the 5-CSRTT (Christakou et al., 2004; Feja et al., 2014). In this regard, the current behavioral findings contrast these results from lesion studies. In a similar vein, subthalamic nucleus (STN) DBS effects are different from STN lesions regarding impulsive action in the 5-CSRTT (Baunez and Robbins, 1997; Baunez et al., 2007). Together, this confirms that DBS exerts more complex effects rather than local inhibition or excitation of neuronal populations *per se*. As such, this is in line with accumulating evidence from human and animal work showing that NA DBS exhibits its effects by altering frontostriatal connectivity via antidromic activity (McCracken and Grace, 2007, 2009; Van Dijk et al., 2011; Do-Monte et al., 2013; Figee et al., 2013; Sesia et al., 2014).

Interestingly, NAcore DBS was found to affect premature and perseverative responses in the 5-CSRTT in a distinct manner. Premature responding in the 5-CSRTT is thought to reflect deficits in inhibitory control of highly prepotent responses when anticipating reward (Evenden, 1999), whereas perseveration after correct choice is thought to reflect compulsivity by action continuation despite reward presentation (Robbins, 2002; Robbins et al., 2012). Strikingly, it has been shown in OCD patients that increase of voltage was found to result in decreased compulsivity, yet at the same time increased impulsivity (Luigjes et al., 2011). These differential effects of NAcore DBS on both behaviors and the lack of correlation between perseverative and premature responding suggest that there is a (partly) distinct underlying neural circuitry, which might be differentially affected by DBS. Indeed, frontostriatal brain regions mostly appear to be involved in either impulsive behavior or compulsive behavior. For instance, the NAcore is suggested to modulate inhibitory control dependent on the reward outcome or success on previous trials (Christakou et al., 2004), which fits with observations that NA neurons code reward expectancy (Apicella et al., 1991; Schultz et al., 1992; Bowman et al., 1996) and respond differentially to cues that predict reward or no reward (Bowman et al., 1996; Donnelly et al., 2014). In addition, we recently found that optogenetic inhibition of the medial prefrontal cortex (mPFC) selectively increased premature responding and not perseverative responding in the 5-CSRTT (Luchicchi et al., 2016). Pharmacological studies also suggest that within these regions there are distinct neurochemical processes that are involved in either premature responding or action perseveration (Carli et al., 2006). Thus, the current distinct effects of DBS on premature and perseverative responding might result from differential activation of the frontostriatal circuitry, in line with the current hypotheses on the mechanism of action of DBS (McIntyre and Hahn, 2010).

Task challenges in the 5-CSRTT such as variable ITI durations result in reduced temporal predictability of the cue, thereby increasing the demand on inhibiting inappropriate responding. Effects under variable ITI conditions are therefore more likely to reflect state impulsivity rather than trait impulsivity. Here, in contrast to fixed ITI conditions, NAcore DBS did not alter premature responding under variable ITI conditions in HI and LI rats. These findings are consistent with previous data in a simple reaction time paradigm (Sesia et al., 2010). This suggests that NAcore DBS specifically affects pre-existing trait impulsivity and not state impulsivity. Notably, variable ITI duration was found to reveal specific effects of NAcore DBS on perseverative responding in HI animals that were not observed under standard fixed ITI task conditions. As expected, in the current study an acute amphetamine challenge reliably increased premature responses in the 5-CSRTT (Cole and Robbins, 1989; Van Gaalen et al., 2006a; Pattij et al., 2007; Baarendse and Vanderschuren, 2012). The fact that NAcore DBS was ineffective in reducing amphetamine-induced impulsivity supports the notion that NAcore DBS specifically targets trait and not state impulsive behavior.

Several lines of evidence suggest that high and low impulsive rats differ neurochemically, for example regarding prefrontal and striatal dopamine functioning (Dalley et al., 2007; Diergaarde et al., 2008; Besson et al., 2010, 2013; Loos et al., 2010; Ohno et al., 2012; Jupp et al., 2013; Moreno et al., 2013). These data are paralleled by clinical evidence, showing that impulsive individuals have decreased dopamine release (Oswald et al., 2007; Buckholtz et al., 2010) and decreased availability of dopamine D2/3 receptors in the striatum (Lee et al., 2009; Ghahremani et al., 2012). Therefore, it is conceivable that the observed baseline-dependent effects of NAcore DBS on impulsive behavior in the current study emerge from underlying neurobiological differences in frontostriatal circuits. Indeed, this notion fits with recent clinical work in OCD patients, demonstrating that DBS reduces OCD symptomatology by restoring NA-PFC network activity, the latter which strongly related to OCD symptom severity (Figee et al., 2013). Similarly, in healthy volunteers non-invasive transcranial direct current stimulation of the dorsolateral PFC was found to modulate impulsivity in a baseline-dependent manner (Shen et al., 2016). Our data align well with these recent observations in humans.

From a clinical perspective, maladaptive trait impulsivity is strongly related to compulsive drug seeking in SUD and compulsive behavior in OCD (Arzeno Ferrao et al., 2006; Penades et al., 2007; Perry and Carroll, 2008; Winstanley et al., 2010; Pattij and De Vries, 2013; Figee et al., 2016). Moreover, it has been shown that individual differences in baseline impulsivity result in differential treatment response (Schmaal et al., 2012; Joos et al., 2013). Our current data suggest that high trait impulsivity can be reversed by DBS, which could ultimately explain the beneficial effects of DBS on impulsivity-related disorders such as SUD and OCD. As yet, there is only limited clinical evidence that has directly addressed the effects of DBS on impulsivity. To the best of our knowledge, there is only a single clinical case report describing direct effects of DBS on measures of impulsivity in SUD. In this report, NA DBS for severe alcohol dependence was found to alter activity in brain networks of inhibitory control, leading to improved inhibitory control in a gambling task (Heldmann et al., 2012). This observation indeed suggests that improvement of impulsivity contributes to the clinical efficacy of DBS in SUD. Vice versa, an important consideration of our current data is that NA DBS treatment for disorders that are not accompanied by maladaptive impulsivity could have unintended side-effects and increase impulsive behavior. Another consideration regarding our animal work relates to the neuroanatomical position of the DBS electrodes in the NA in humans and rats. In general, in humans the most effective stimulation site in the NA is the border of the lateral accumbens and the capsula interna (Valencia-Alfonso et al., 2012; Figee et al., 2013). Although, histochemically the lateral part of the accumbens displays similarities to the NAcore of the rat (Voorn et al., 1996), it is not known whether this region is a functional homolog of the rodent NAcore.

Future research on the neurobiological mechanism underlying the baseline-dependent effects of NA DBS in rats and if possible humans, on measures of impulsivity is warranted. In addition, it would be of interest to extend the effects of NAcore DBS in rats to other forms of impulsive behavior, such as response inhibition, another important measure of impulsivity related to SUD and OCD (Grant and Chamberlain, 2014; Jupp and Dalley, 2014; Van Velzen et al., 2014). Particularly, since multiple studies have revealed that different forms of impulsive behavior emerge from (partly) distinct underlying neurobiological pathways (Pattij and Vanderschuren, 2008; Dalley et al., 2011; Bari and Robbins, 2013).

Taken together, we found evidence that NAcore DBS exerts baseline-dependent effects on impulsive action and impulsive choice. As such, the current findings extend our understanding of mechanisms of DBS and the way how DBS may exert clinical effects in psychiatric disorders with maladaptive impulsivity including SUD and OCD.

## Author contributions

TP, TD, and MS designed the study. MS performed surgeries, behavioral experiments and perfusions. BB, MG, and TM assisted in the training, behavioral experiments and perfusions. MG assisted with surgeries. MS, BB, MG, and TM analyzed the behavioral data. MS, TD, and TP wrote the manuscript with input from BB and DD.

## Funding

This research was funded by ZonMW grant 31160204.

### Conflict of interest statement

The authors declare that the research was conducted in the absence of any commercial or financial relationships that could be construed as a potential conflict of interest.
